# Corrigendum

**DOI:** 10.1002/ehf2.14007

**Published:** 2022-06-06

In the original published version of the paper of Tahara *et al*.^1^ the authors noticed errors in ‘False positives, false negatives, and inconsistencies between ^99m^Tc‐PYP scintigraphy and biopsy findings’ section and *Tables* *2* *and* *3*.

On page 257 of the article, in the ‘False positives, false negatives, and inconsistencies between ^99m^Tc‐PYP scintigraphy and biopsy findings’ section, the cases of ‘rib fractures, valvular/annular calcifications, and recent myocardial infarction’ need to be regarded as false positive instead of false negative. The revised main text should thus read as follows:

‘False‐positive cases in ^99m^Tc‐PYP scintigraphy for ATTR‐CM may include Grade 2 or 3 myocardial uptake; recent myocardial infarction (<4 weeks) ^10^; rib fractures ^10^ ; valvular/annular calcifications ^10^ ; AL amyloidosis^34,54,69–73^; apolipoprotein AI amyloidosis (AApoAI), apolipoprotein AII amyloidosis (AApoAII), apolipoprotein A‐IV amyloidosis (ApoAAIV), or β2‐microglobulin amyloidosis (Aβ2M)^10^; hypertrophic cardiomyopathy^74,75^; hydroxychloroquine toxicity^76^; and cardiac blood pools.^54^ Case reports on false‐positive myocardial uptake following intravenous iron injections have been published for ^99m^Tc‐DPD and ^99m^Tc‐HMDP scintigraphy.^77,78^ In contrast, Grade 0 or 1 myocardial uptake, an insufficient amount of amyloid deposits, ^79^ delayed or premature acquisition in ^99m^ Tc‐PYP scintigraphy, ^10^ ATTRv‐CM with a low sensitivity for ^99m^Tc‐labelled bone radiotracer scintigraphy (Ser77Tyr or Phe64Leu mutation),^10,80^ and initial diagnosis of cardiac pools with myocardial deposits^81^ are examples of possible false‐negative cases (*Table* *2*).’

Similarly, the current *Table* *2* (page 258). ‘Rib fractures and valvular/annular calcifications’ and ‘Recent myocardial infarction (<4 weeks)’ should be removed from false negative causes and combined into false‐positive causes. The revised *Table* *2* should read as follows:

**Table 2:** Typical false‐positive and false‐negative cases in planar ^99m^Tc‐PYP scintigraphy for the diagnosis of ATTR‐CM

Planar ^99m^Tc‐PYP scintigraphy results	Potential causes of false results
False positive	Recent myocardial infarction (<4 weeks) Rib fractures and valvular/annular calcifications AL amyloidosis AApoAI, AApoAII, AApoAIV, and Aβ2M Hypertrophic cardiomyopathy Hydroxychloroquine toxicity Cardiac blood pool Intravenous iron injections
False negative	Insufficient amount of amyloid deposits Delayed or premature acquisition in ^99m^ Tc‐PYP scintigraphy ATTRv‐CM with a low sensitivity in scintigraphy (Ser77Tyr or Phe64Leu mutation) Initial diagnosis of cardiac pools with myocardial deposits

In addition, the current *Table* *3* (page 258). The Ser77Tyr mutation should be added to ‘ATTRv‐CM with a low sensitivity in ^99m^Tc‐PYP scintigraphy (Phe64Leu mutation)’ for consistency with *Table* *2*. The revised *Table* *3* should thus read as follows:

**Table 3:** Potential causes of inconsistencies between planar ^99m^Tc‐PYP scintigraphy and biopsy findings in suspected ATTR‐CM

Planar ^99m^Tc‐PYP scintigraphy	Biopsy	Potential causes of inconsistencies
Positive	Negative	Sampling errors in biopsy Errors in scintigraphy imaging time or evaluation Errors in pathological diagnosis Cardiac pools Deposit of proteins other than transthyretin (e.g. AL)
Negative	Positive	Errors in scintigraphy evaluation Errors in pathological diagnosis Deposit of proteins other than transthyretin (e.g. AL) ATTRv‐CM with a low sensitivity in ^99m^Tc‐PYP scintigraphy (Ser77Tyr or Phe64Leu mutation)

These errors occurred due to an inadvertent oversight and misinterpretation of source data. This change will not impact any other part of the manuscript including the abstract, and it does not change the data interpretation or conclusions in any way.
